# Prognostic value of the hemoglobin, albumin, lymphocyte, and platelet score in nonmetastatic renal cell carcinoma after nephrectomy

**DOI:** 10.1097/MD.0000000000051294

**Published:** 2026-10-23

**Authors:** Zheng Huang, Dan Chen

**Affiliations:** a Department of Urology, Chengdu Seventh People’s Hospital, Affiliated Cancer Hospital of Chengdu Medical College, Chengdu, China.

**Keywords:** Biomarker, HALP Score, Nephrectomy, Renal Cell Carcinoma, Survival Analysis

## Abstract

The hemoglobin, albumin, lymphocytes, and platelets (HALP) score is a comprehensive indicator that reflects the body’s systemic nutritional and immune-inflammatory status. Previous studies have found HALP score to be a strong predictor of several cancers. However, its predictive efficacy in nonmetastatic renal cell carcinoma (RCC) has been poorly studied. Patients diagnosed with nonmetastatic RCC who underwent nephrectomy at West China Hospital of Sichuan University between 2009 and 2013 were included in this study. The relationship between the HALP score and overall survival (OS) was initially observed using restricted cubic splines (RCS), with the cutoff value determined through the Maximally Selected Log-Rank Statistic. Logistic regression was utilized to explore the correlation between the HALP score and clinicopathological factors. Propensity score matching (PSM) was then employed to balance baseline characteristics of the groups. Finally, Cox regression analysis was conducted to assess the independent prognostic value of the HALP score. A total of 1164 patients were included. The cutoff score for the HALP score was determined to be 38.3. Logistic regression found that a lower HALP score was associated with poor clinicopathological factors, specifically worse Eastern Cooperative Oncology Group (ECOG) status, larger tumor size, advanced pT stage, and high Fuhrman grade. After PSM, Cox regression analysis confirmed the independent predictive value of a higher HALP score for better OS (HR = 0.50, 95% CI 0.34–0.75, *P* < .001). The HALP score emerges as a vital prognostic tool for assessing OS in RCC patients post-nephrectomy, effectively encapsulating the combined effects of systemic nutritional and immune-inflammatory factors.

## 1. Introduction

Renal cell carcinoma (RCC) is one of the most common malignant tumors. In 2020, an estimated 431,288 new cases were reported worldwide, accounting for 2.2% of all new cancer cases.^[1]^ The incidence of kidney cancer has been continually rising over the past few decades, largely due to the widespread use of ultrasonography and computed tomography (CT) scans.^[2]^ Nephrectomy remains the primary treatment for localized RCC. However, despite most patients being diagnosed in the early stages of the disease, approximately 20% to 30% of those with localized RCC progress to metastatic RCC after nephrectomy, requiring systemic therapy and having a poor prognosis.^[3,4]^ Therefore, accurate prognostic markers and predictive models are needed to clearly define patient clinical outcomes and guide treatment. Prognostic factors for RCC include anatomical, histological, clinical, and molecular factors.^[3]^ The TNM staging system encapsulates the anatomical factors, while the most significant histological factor, the Fuhrman grade, is gradually being replaced by the WHO/ISUP grading classification.^[5]^ Numerous molecular prognostic factors, such as PD-L1,^[6]^ and PTEN cell cycle,^[7]^ have been explored in RCC, but few have undergone extensive external validation, limiting their clinical application. Clinical factors include patient performance status, symptoms, and various laboratory and derivative indices. In recent years, the therapeutic landscape of RCC has been substantially transformed by the development of novel systemic therapies, particularly immune checkpoint inhibitors and targeted therapies, which have improved outcomes for patients with advanced disease.^[8,9]^ Moreover, ongoing advances in treatment strategies, including combination regimens and biomarker-guided approaches, have further emphasized the clinical heterogeneity of RCC and the need for complementary prognostic tools to refine risk stratification.^[10–12]^

Laboratory and derivative indices are diverse, aiding in objectively evaluating various patient states, and hold the potential to more accurately predict patient survival. Previous studies have shown that indices derived from laboratory indicators can objectively assess the nutritional status of RCC patients, such as the prognostic nutritional index (PNI),^[13]^ and systemic inflammatory status, such as various systemic inflammation indices,^[14]^ proving to be related to patient prognosis. However, prognosis is a multifaceted outcome influenced by various factors, among which the host’s systemic nutritional and immune-inflammatory status play important roles. Compared to individual factors, integrating these aspects might provide a more detailed prognosis assessment. The hemoglobin albumin lymphocyte and platelet (HALP) score, as a composite index, reflects systemic nutritional and immune-inflammatory status by integrating hemoglobin, albumin, lymphocyte, and platelet counts. Previous studies have found the HALP score to be a robust predictor in several cancers, including hepatocellular carcinoma,^[15]^ esophageal squamous cell carcinoma,^[16]^ and gastrointestinal stromal tumors.^[17]^ However, its predictive efficacy in nonmetastatic RCC has been rarely studied. In light of this, our study aims to investigate the prognostic significance of the HALP score in the context of nonmetastatic RCC.

## 2. Patients and methods

### 2.1. Patients

We retrospectively collected clinical data from patients who underwent nephrectomy at West China Hospital of Sichuan University between 2009 and 2013. The inclusion criteria were as follows: age 18 years or older; postoperative pathological confirmation of RCC; pathological staging of pTanyN0M0. Patient follow-up was conducted through outpatient visits and telephone calls. The primary endpoint of this study was overall survival (OS), defined as the time interval from nephrectomy to all-cause mortality or the last follow-up.

Clinical information for patients included age, gender, Eastern Cooperative Oncology Group (ECOG) performance status, surgical approach, tumor laterality, size, histological subtype, Fuhrman grade, and results of hemoglobin, albumin, lymphocyte count, and platelet count taken within one week before nephrectomy. Tumor size was based on the maximum diameter reported in the pathology report. All patients underwent chest and abdominal CT scans before surgery to rule out metastasis, and those suspected of lymph node metastasis underwent lymph node dissection during surgery. Pathological staging was based on the 8th edition of the AJCC TNM staging system. All included patients underwent routine blood and biochemical tests within one week before surgery, from which we obtained results for hemoglobin, albumin, lymphocyte count, and platelet count, and calculated the HALP score. When multiple tests were performed preoperatively, the one closest to the surgery was adopted. The formula for the HALP score is as follows: hemoglobin level (g/L) × albumin level (g/L) × lymphocyte (/L)/platelet count (/L).

This research was approved by the ethics committee of the West China Hospital, Sichuan University (2023-463). All methods were performed in accordance with the ethical standards set by the ethics committee of West China Hospital, Sichuan University, as well as the Declaration of Helsinki. Due to the retrospective nature of the study, informed consent was waived by the ethics committee of West China Hospital, Sichuan University.

### 2.2. Statistical analysis

Firstly, the potential nonlinear relationship between changes in the HALP score and OS was examined using a Cox regression model with restricted cubic splines (RCS). Knots between 3 and 7 were tested, and the model with the lowest Akaike Information Criterion (AIC) was selected for RCS. When interpreting the results of the RCS analysis, the median value of the predictor variable was chosen as the reference. Subsequently, the Maximally Selected Log-Rank Statistic was utilized to determine an optimal cutoff value that would create the most significant survival difference between the two divided groups. To explore the correlation between the HALP score and clinicopathological factors, continuous variables such as age and tumor size were categorized based on the median age of patients and median tumor size. Age was divided into < 55 and ≥ 55 years, and tumor size into < 4.5 and ≥ 4.5 cm. Logistic regression was then employed to investigate the correlation between the HALP score and pathological clinical factors, with variables significant in univariate logistic regression included in subsequent multivariate logistic regression. Thereafter, the Kaplan–Meier (K-M) curve was used to estimate the OS of patients, and the Log-rank test was applied to compare the significance of survival differences between the 2 groups. To minimize potential bias caused by differences in baseline characteristics between patients with different HALP scores, 1:1 propensity score matching (PSM) was performed. The propensity score was estimated using logistic regression including age, sex, ECOG performance status, surgical method, tumor side, tumor size, pT stage, histological subtype, and Fuhrman grade. Patients were matched using nearest-neighbor matching without replacement at a 1:1 ratio, with a caliper width of 0.2 of the standard deviation of the logit of the propensity score. Covariate balance before and after matching was assessed using standardized mean differences (SMDs), with an SMD < 0.1 considered indicative of adequate balance. After matching, Kaplan–Meier analysis and Log-rank tests were repeated to compare OS between groups. Subgroup analysis based on predetermined variables (age, sex, ECOG status, size, T stage, histology, and Fuhrman grade) was also performed. Following this, Cox regression was utilized to ascertain the independent predictive efficacy of the HALP score, with variables significant in univariate Cox regression included in subsequent multivariate Cox regression to determine their prognostic value. All statistical analyses were performed using R software (version 4.3.1; R Foundation for Statistical Computing, Vienna, Austria). A two-sided *P*-value < .05 was considered statistically significant.

## 3. Results

### 3.1. Baseline characteristics

A total of 1164 patients were included in the study. The majority of participants were male (61.3%), and most had an ECOG status of 0 (68.9%). Radical nephrectomy was the most common surgical method (69.3%), with tumor laterality almost equally distributed between left (50.3%) and right (49.7%). Most participants had T1-T2 stage tumors (90.6%) and clear cell RCC (88.1%). Additionally, the mean values of hemoglobin, albumin, lymphocyte count, platelet count, and HALP score were 136 ± 20 g/L, 42.6 ± 4.0 g/L, 1.63 ± 0.54 × 10^9^/L, 180 ± 68 × 10^9^/L, and 61 ± 38, respectively. Baseline characteristics of patients are shown in Table 1.

**Table 1:** Patient demographics and baseline characteristics.

Characteristic	N = 1,164*
Age, year	
Mean ± SD	55 ± 13
Sex	
Male	714 (61.3%)
Female	450 (38.7%)
ECOG status	
0	802 (68.9%)
≥1	362 (31.1%)
Surgical method	
Partial nephrectomy	357 (30.7%)
Radical nephrectomy	807 (69.3%)
Tumor side	
Left	586 (50.3%)
Right	578 (49.7%)
Size, cm	
Mean ± SD	4.92 ± 2.56
pT stage	
T1-T2	1055 (90.6%)
T3-T4	109 (9.4%)
Histology	
ccRCC	1026 (88.1%)
Non-ccRCC	138 (11.9%)
Fuhrman grade	
Grade 1–2	646 (55.5%)
Grade 3–4	518 (44.5%)
Hemoglobin, g/L	
Mean ± SD	136 ± 20
Albumin, g/L	
Mean ± SD	42.6 ± 4.0
Lymphocyte count, 10^9^/L	
Mean ± SD	1.63 ± 0.54
Platelet count, 10^9^/L	
Mean ± SD	180 ± 68
HALP score	
Mean ± SD	61 ± 38

ccRCC = Clear Cell Renal Cell Carcinoma, ECOG = Eastern Cooperative Oncology Group, HALP score = Hemoglobin, Albumin, Lymphocyte, Platelet score, SD = Standard Deviation.

* n (%).

### 3.2. HALP score cutoff

Using RCS with 3 knots, RCS analysis indicated a nonlinear association between HALP score and OS (*P* < .001, *P*-Nonlinear < .001, Fig. 1), with OS improving as the HALP score increased. Using the Maximally Selected Log-Rank Statistic, we set the cutoff value at 38.3, which resulted in the most significant survival difference between the 2 groups (Fig. 2).

**Figure 1.**
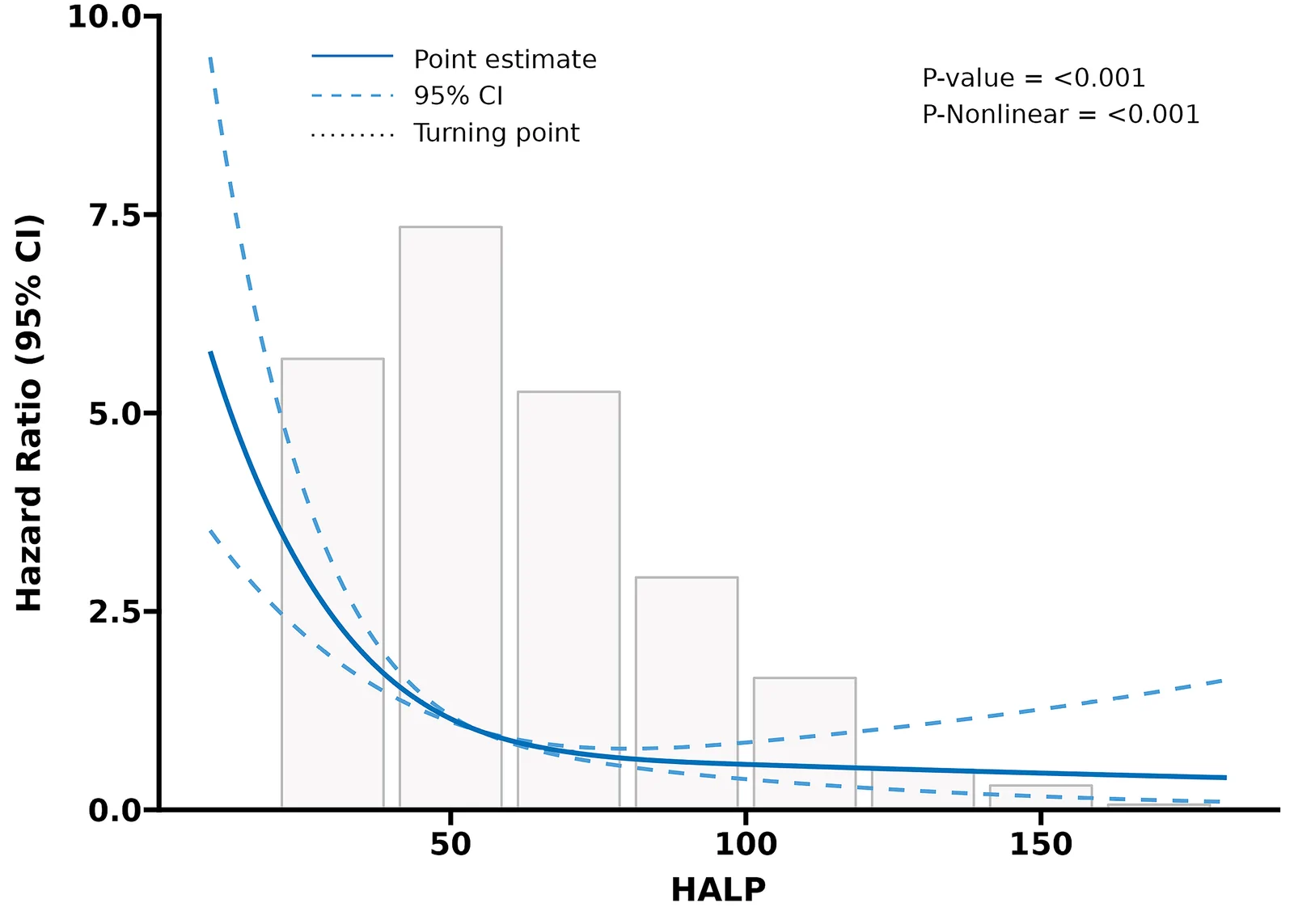
A nonlinear relationship between HALP score and overall survival in patients with nonmetastatic renal cell carcinoma who underwent nephrectomy.

**Figure 2.**
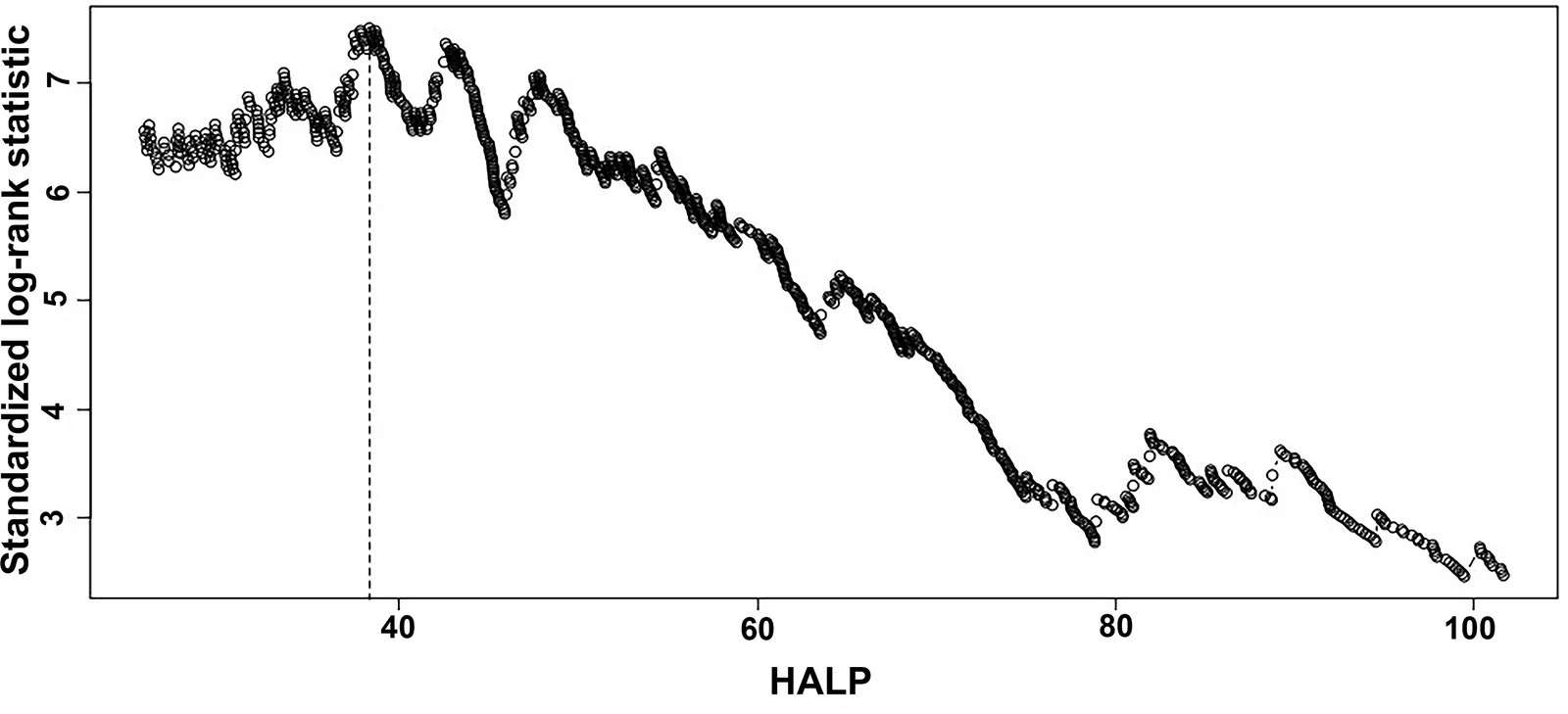
The maximally selected log-rank statistic determined the optimal cutoff values for HALP score.

### 3.3. Correlation between HALP and clinicopathological factors

In univariate logistic regression, age, sex, ECOG status, size, pT stage, and Fuhrman grade were identified as potential predictors for the HALP score and were included in the subsequent multivariate logistic regression. Ultimately, in multivariate logistic regression, females (OR = 2.22, 95% CI 1.68–2.93, *P* < .001), worse ECOG status (OR = 1.41, 95% CI 1.03–1.91, *P* = .030), larger tumor size (OR = 1.53, 95% CI 1.15–2.03, *P* = .003), advanced pT stage (OR = 2.20, 95% CI 1.43–3.37, *P* < .001), and higher Fuhrman grade (OR = 1.41, 95% CI 1.06–1.88, *P* = .017) were found to be more likely to have lower HALP scores. The results of the logistic regression are presented in Table 2.

**Table 2:** Univariate and multivariate analysis of the association between HALP score and patient characteristics.

Characteristic	UnivWariate		Multivariate	
	OR (95% CI)	*P*-value	OR (95% CI)	*P*-value
Age				
< 55	Ref		Ref	
≥ 55	1.31 (1.00–1.71)	.047	1.06 (0.78–1.42)	.716
Sex				
Male	Ref		Ref	
Female	2.00 (1.53–2.61)	<.001	2.22 (1.68–2.93)	<.001
ECOG status				
0	Ref		Ref	
>= 1	1.57 (1.19–2.07)	.001	1.41 (1.03–1.91)	.030
Tumor side				
Left	Ref			
Right	0.84 (0.64–1.10)	.200		
Size				
< 4.5	Ref		Ref	
≥ 4.5	1.76 (1.34–2.30)	<.001	1.53 (1.15–2.03)	.003
pT stage				
T1-T2	Ref		Ref	
T3-T4	2.60 (1.73–3.89)	<.001	2.20 (1.43–3.37)	<.001
Histology				
ccRCC	Ref			
Non-ccRCC	1.06 (0.70–1.57)	.792		
Fuhrman grade				
Grade 1–2	Ref		Ref	
Grade 3–4	1.60 (1.23–2.09)	<.001	1.41 (1.06–1.88)	.017

ccRCC = Clear Cell Renal Cell Carcinoma, ECOG = Eastern Cooperative Oncology Group, HALP score = Hemoglobin, Albumin, Lymphocyte, Platelet score.

### 3.4. Overall survival

Before PSM, there were 871 patients with a HALP score ≥ 38.3 and 293 patients with a HALP score < 38.3. There was a significant baseline difference between the 2 groups in terms of age (*P* = .047), sex (*P* < .001), ECOG performance status (*P* = .001), surgical method (*P* < .001), tumor size (*P* < .001), pT stage (*P* < .001), and Fuhrman grade (*P* < .001), as shown in Table 3. After PSM, a total of 293 patients with HALP scores < 38.3 and ≥ 38.3 each were included. The baseline characteristics were well balanced between the 2 groups, with all SMD values below 0.1 after matching (Table 3). Before PSM, patients with a HALP score of ≥ 38.3 had better OS compared to those with HALP < 38.3 (HR = 0.29, 95% CI 0.21–0.41, *P* < .001, Fig. 3A). The 12-, 36-, and 60-month OS rates for patients with a HALP score < 38.3 were 94.9% (95% CI, 92.4–97.4), 86.3% (95% CI, 82.5–90.4), and 79.3% (95% CI, 74.7–84.1), respectively, while the 12-, 36-, and 60-month OS rates for patients with a HALP score ≥ 38.3 were 99.5% (95% CI, 99.1–100.0), 96.4% (95% CI, 95.2–97.7), and 93.9% (95% CI, 92.4–95.6), respectively (Table 4). After PSM, compared to those with HALP < 38.3, patients with HALP ≥ 38.3 still had better but moderated OS (HR = 0.51, 95% CI 0.34–0.75, *P* < .001, Fig. 3B). The 12-, 36-, and 60-month OS rates for patients with a HALP score < 38.3 were 88.7% (95% CI, 85.2–92.4), 80.8% (95% CI, 76.4–85.5), and 77.6% (95% CI, 72.9–82.5), respectively, while the 12-, 36-, and 60-month OS rates for patients with a HALP score ≥ 38.3 were 94.5% (95% CI, 92–97.2), 91.1% (95% CI, 87.9–94.4), and 89.0% (95% CI, 85.4–92.6), respectively (Table 4). As shown in Figure 4, in subgroup analysis, patients with HALP ≥ 38.3 resulted in better OS compared with those with HALP < 38.3 in all pre-determined characteristics (all HR < 1, *P* < .05), except in non-clear cell RCC (non-ccRCC) which was not significant (HR = 0.76, 95% CI, 0.25–2.31, *P* = .622).

**Table 3:** Patient demographics and baseline characteristics before and after propensity score matching.

Characteristic	Before PSM				After PSM			
	≥ 38.3, N = 871*	< 38.3, N = 293*	*P*-value**	SMD	≥ 38.3, N = 293*	< 38.3, N = 293*	*P*-value**	SMD
Age, year			.047	0.135			.559	0.048
< 55	439 (50.4%)	128 (43.7%)			121 (41.3%)	128 (43.7%)		
≥ 55	432 (49.6%)	165 (56.3%)			172 (58.7%)	165 (56.3%)		
Sex			<.001	0.339			.869	0.014
Male	571 (65.6%)	143 (48.8%)			141 (48.1%)	143 (48.8%)		
Female	300 (34.4%)	150 (51.2%)			152 (51.9%)	150 (51.2%)		
ECOG status			.001	0.211			.733	0.028
0	622 (71.4%)	180 (61.4%)			184 (62.8%)	180 (61.4%)		
>= 1	249 (28.6%)	113 (38.6%)			109 (37.2%)	113 (38.6%)		
Surgical method			<.001	0.253			.921	0.008
RN	579 (66.5%)	228 (77.8%)			227 (77.5%)	228 (77.8%)		
PN	292 (33.5%)	65 (22.2%)			66 (22.5%)	65 (22.2%)		
Tumor side			.2	0.087			.244	0.096
Left	429 (49.3%)	157 (53.6%)			171 (58.4%)	157 (53.6%)		
Right	442 (50.7%)	136 (46.4%)			122 (41.6%)	136 (46.4%)		
Size, cm			<.001	0.279			.736	0.028
< 4.5	466 (53.5%)	116 (39.6%)			120 (41.0%)	116 (39.6%)		
≥ 4.5	405 (46.5%)	177 (60.4%)			173 (59.0%)	177 (60.4%)		
pT stage			<.001	0.292			.42	0.067
T1-T2	810 (93.0%)	245 (83.6%)			252 (86.0%)	245 (83.6%)		
T3-T4	61 (7.0%)	48 (16.4%)			41 (14.0%)	48 (16.4%)		
Histology			.792	0.018			.357	0.076
ccRCC	769 (88.3%)	257 (87.7%)			264 (90.1%)	257 (87.7%)		
Non-ccRCC	102 (11.7%)	36 (12.3%)			29 (9.9%)	36 (12.3%)		
Fuhrman grade			<.001	0.234			.679	0.034
Grade 1–2	509 (58.4%)	137 (46.8%)			142 (48.5%)	137 (46.8%)		
Grade 3–4	362 (41.6%)	156 (53.2%)			151 (51.5%)	156 (53.2%)		

cRCC = Clear Cell Renal Cell Carcinoma, ECOG = Eastern Cooperative Oncology Group, PN = Partial Nephrectomy, PSM = Propensity Score Matching, RN = Radical Nephrectomy, SMD = Standardized Mean Difference.

* n (%).

** Pearson’s Chi-squared test.

**Table 4:** Kaplan–Meier estimates of 12-, 36-, and 60-month overall survival in patients with nonmetastatic renal cell carcinoma undergoing nephrectomy with a HALP score < 38.3 and ≥ 38.3.

Characteristic	12-month	36-month	60-month	*P*-value*
Before PSM	.			
<38.3	94.9% (92.4%, 97.4%)	86.3% (82.5%, 90.4%)	79.3% (74.7%, 84.1%)	<.001
≥38.3	99.5% (99.1%, 100.0%)	96.4% (95.2%, 97.7%)	93.9% (92.4%, 95.6%)	
After PSM				
<38.3	88.7% (85.2%, 92.4%)	80.8% (76.4%, 85.5%)	77.6% (72.9%, 82.5%)	<.001
≥38.3	94.5% (92.0%, 97.2%)	91.1% (87.9%, 94.4%)	89.0% (85.4%, 92.6%)	

OS = overall survival, PSM = propensity score matching.

* Log-rank test.

**Figure 3.**
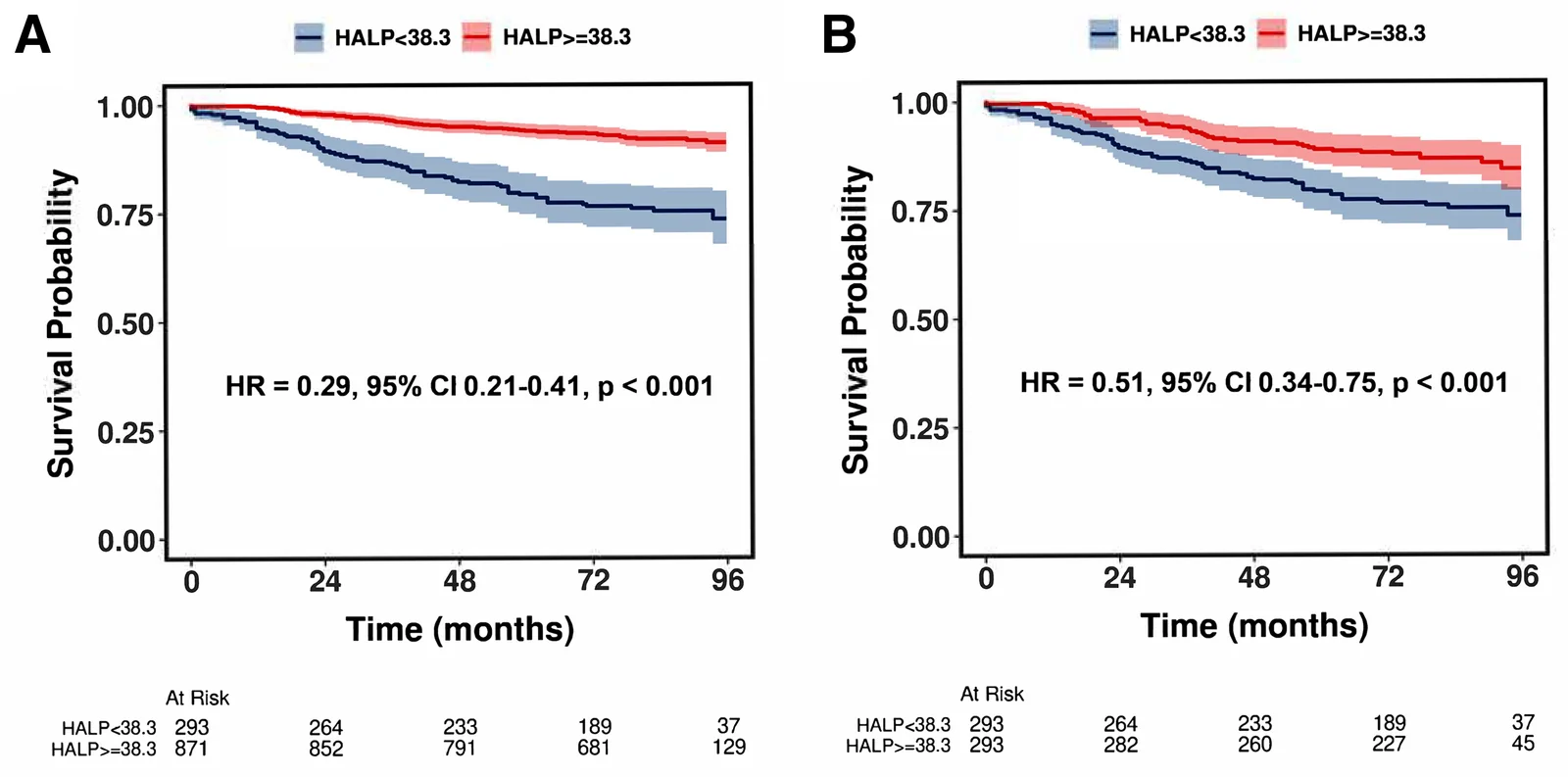
Kaplan–Meier overall survival curves before (A) and after (B) propensity matching score.

**Figure 4.**
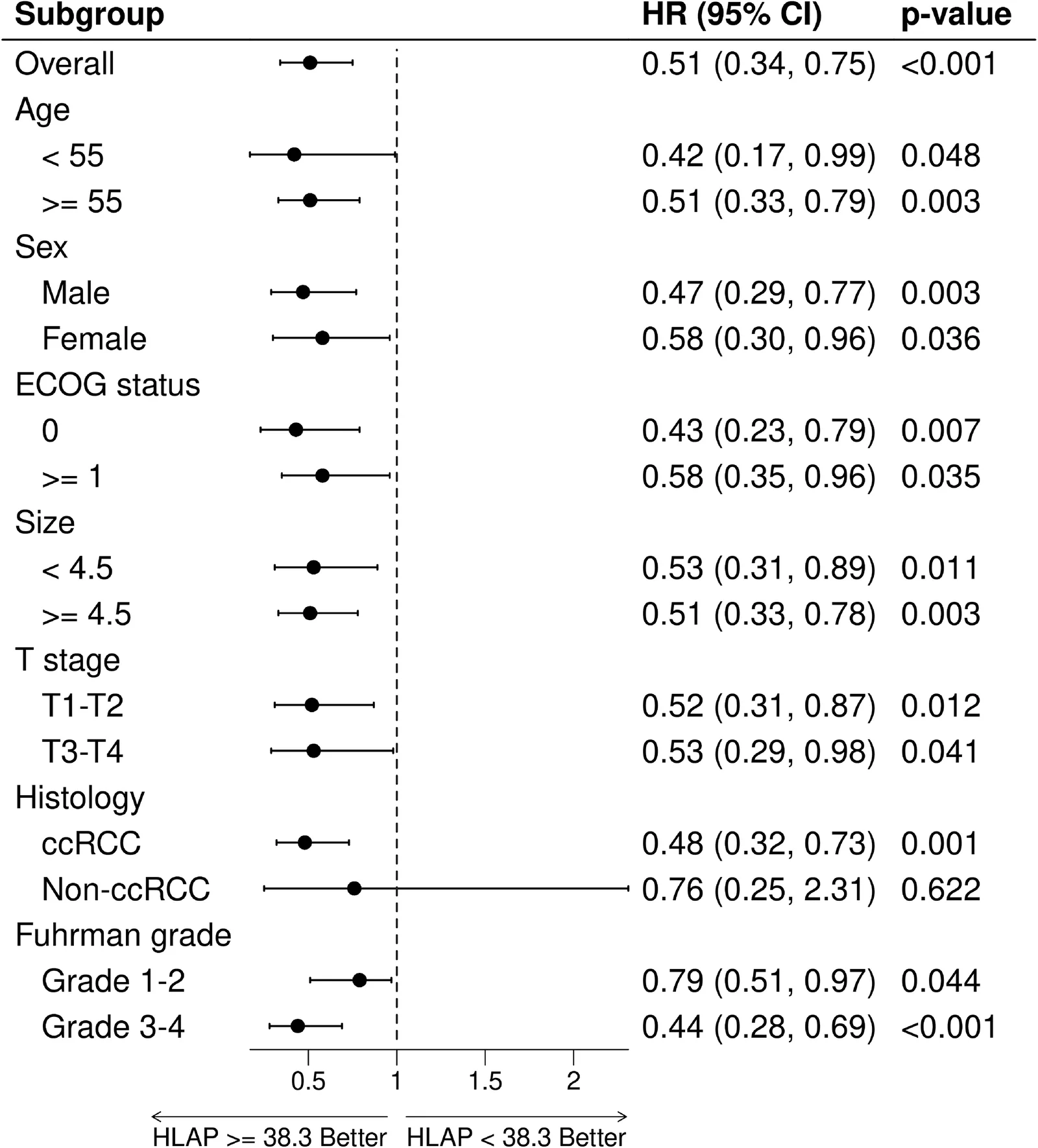
Forest plot for subgroup analysis.

As shown in Table 5, in univariate Cox regression analysis, age, sex, ECOG status, surgical method, size, pT stage, Fuhrman grade, and HALP score were identified as potential independent predictors for OS (all *P* < .05). In multivariate analysis, worse ECOG performance status (ECOG status ≥ 1 vs ECOG status 0, HR = 1.89, 95% CI 1.26–2.82, *P* = .002), larger tumor size (≥4.5 vs <4.5 cm, HR = 2.39, 95% CI 1.35–4.23, *P* = .003), advanced pT stage (pT 3–4 vs pT 1–2, HR = 3.80, 95% CI 2.52–5.71, *P* < .001), and higher Fuhrman grade (Grade 3–4 vs Grade 1–2, HR = 1.78, 95% CI 1.09–2.92, *P* = .021) were associated with poorer prognosis, while a higher HALP score (≥38.3 vs <38.3, HR = 0.50, 95% CI 0.34–0.75, *P* < .001) independently predicted better OS.

**Table 5:** Univariate and multivariate Cox regression for overall survival.

Characteristic	Univariate		Multivariate	
	HR (95% CI)	*P*-value	HR (95% CI)	*P*-value
Age				
< 55	Ref		Ref	
≥ 55	2.89 (1.83–4.54)	<.001	1.57 (0.96–2.57)	.071
Sex				
Male	Ref		Ref	
Female	0.48 (0.32–0.70)	<.001	0.74 (0.49–1.11)	.147
ECOG status				
0	Ref		Ref	
≥ 1	2.50 (1.71–3.66)	<.001	1.89 (1.26–2.82)	.002
Surgical method				
Radical nephrectomy	Ref		Ref	
Partial nephrectomy	0.22 (0.10–0.47)	<.001	0.71 (0.30–1.66)	.424
Tumor side				
Left	Ref			
Right	1.08 (0.74–1.58)	.683		
Size				
< 4.5 cm	Ref		Ref	
≥ 4.5 cm	4.20 (2.50–7.05)	<.001	2.39 (1.35–4.23)	.003
T stage				
T1-T2	Ref		Ref	
T3-T4	5.91 (4.03–8.67)	<.001	3.80 (2.52–5.71)	<.001
Histology				
ccRCC	Ref			
Non-ccRCC	1.13 (0.63–2.02)	.674		
Fuhrman grade				
Grade 1–2	Ref		Ref	
Grade 3–4	3.73 (2.37–5.88)	<.001	1.78 (1.09–2.92)	.021
HALP score				
< 38.3	Ref		Ref	
≥ 38.3	0.51 (0.34–0.75)	<.001	0.50 (0.34–0.75)	<.001

ccRCC = clear cell renal cell carcinoma, CI = confidence interval, ECOG = Eastern Cooperative Oncology Group, HALP score = hemoglobin, albumin, lymphocyte, and platelet score, HR = hazard ratio.

## 4. Discussion

Cancer-associated anemia is reported to be present in 30% of cancer patients at diagnosis and is associated with more advanced tumor stages.^[18]^ The causes of cancer-related anemia are multifaceted, including nutritional deficiencies.^[19]^ Serum albumin, a protein produced by the liver and the most abundant protein in human plasma, often reflects nutritional deficiency when its levels are low. Hemoglobin and albumin levels, as indicators of nutritional status and systemic conditions, have been reported to be associated with prognosis in various cancers, including RCC.^[20,21]^ Similarly, as a previous study demonstrated, various systemic inflammation indices, including the platelet-to-lymphocyte ratio, are independent predictors of survival in patients with RCC who have undergone nephrectomy.^[14]^ The rationale behind the platelet-to-lymphocyte ratio predicting patient survival is sound: lymphocytes play a crucial role in host immune responses against tumors, and lymphopenia has been found to be associated with poorer outcomes in RCC.^[22]^ Furthermore, current evidence suggests that platelets can protect tumor cells from immune elimination and promote their stasis in endothelial cells, enhancing tumor invasiveness, thereby facilitating tumor progression and metastasis.^[23,24]^

Given the role of nutritional status and inflammatory response in predicting patient prognosis, combining both should provide a more comprehensive assessment of patient outcomes. The HALP score integrates hemoglobin, albumin, lymphocyte, and platelet counts and has been proposed as an accessible biomarker reflecting systemic nutritional and immune-inflammatory status. In a study of 273 patients with hepatocellular carcinoma who underwent surgery, the preoperative HALP score was an an independent predictor of OS, with a lower HALP score associated with poorer prognosis.^[15]^ Similarly, in another study of 834 patients with esophageal squamous cell carcinoma who underwent radical esophagectomy, a lower HALP score was independently associated with poorer survival.^[16]^ In terms of RCC, Ekinci et al found that a lower HALP score independently predicted poorer OS in metastatic RCC.^[25]^ Similarly, Peng et al in a retrospective study of 1360 patients who underwent nephrectomy for RCC found that a lower HALP score was associated with poorer prognosis, and combining the HALP score with Fuhrman grade and TNM staging could more accurately predict patient prognosis.^[26]^ Beyond HALP, increasing evidence has demonstrated that other host-related immune-nutritional indicators provide additional prognostic information in patients with cancer. These readily available biomarkers reflect the complex interactions between systemic inflammation, nutritional status, and tumor progression, and may complement traditional clinicopathological factors in risk assessment. Recent studies have further highlighted the importance of integrating immune-nutritional characteristics and tumor-related molecular information into contemporary prognostic frameworks.^[27–29]^

However, it’s noteworthy that there were significant baseline characteristic differences between the groups with high and low HALP scores in this study, which could lead to potential bias. In our study, using logistic regression, we found that lower HALP scores significantly correlated with poor prognostic features. Moreover, females were also more likely to have lower HALP scores than males, potentially due to differences in baseline characteristics such as hemoglobin, albumin, lymphocyte count, and platelet count between males and females. Using RCS curves, we found that the HALP score positively correlated with OS (*P* < .001), but this relationship was nonlinear (*P*-Nonlinear < .001). After PSM, which balanced baseline characteristics between the 2 groups, we found that a higher HALP score still correlated with better OS (HR = 0.51, 95% CI 0.34–0.75, *P* < .001), suggesting the potential prognostic value of HALP scores in RCC patients. In addition, subgroup analysis showed that the predictive value of the HALP score was significant in patients with different characteristics, although it did not reach statistical significance in non-ccRCC patients. However, the large confidence interval suggested that the small sample size limited the statistical power. After all, there were only 65 non-ccRCC patients after PSM.

Our study has notable strengths, including the use of RCS to analyze the relationship between HALP scores and patient prognosis. RCS is particularly effective for revealing complex, nonlinear relationships in medical data, providing a clearer understanding of how HALP scores relate to patient outcomes. Additionally, employing PSM helped to minimize bias by balancing the baseline characteristics between different patient groups. However, several limitations should be acknowledged. First, this was a retrospective single-center study, and potential selection bias cannot be completely excluded. Although propensity score matching was performed to reduce baseline imbalance between groups, residual confounding due to unmeasured variables may still exist. Second, the HALP cutoff value of 38.3 was determined using the Maximally Selected Log-Rank Statistic within the same cohort in which its prognostic performance was evaluated. Although this approach identified a threshold with significant prognostic discrimination, the possibility of data-driven optimism should be considered. Therefore, this cutoff value requires validation in independent external cohorts before it can be considered a broadly applicable clinical cutoff. Third, although OS was selected as the primary endpoint, cancer-specific survival and recurrence-free survival data were unavailable, which limited further evaluation of the association between HALP score and disease-specific outcomes. Finally, the moderate sample size and retrospective design may limit the generalizability of our findings to broader and more diverse RCC populations.

## 5. Conclusion

Based on a retrospective study of 1164 patients who underwent nephrectomy for RCC, we found that a lower HALP score was significantly associated with adverse prognostic features and that the HALP score was an independent prognostic factor for survival.

## Acknowledgments

We sincerely appreciate the Department of Urology, West China Hospital, Sichuan University, for providing the clinical data and supporting this study.

## Author contributions

**Conceptualization:** Zheng Huang, Dan Chen.

**Data curation:** Zheng Huang.

**Formal analysis:** Zheng Huang, Dan Chen.

**Investigation:** Zheng Huang, Dan Chen.

**Writing – original draft:** Zheng Huang, Dan Chen.

**Writing – review & editing:** Zheng Huang, Dan Chen.

**Supervision:** Dan Chen.
